# Can Laparoscopic Surgery Reduce Fatigue in Women with Endometriosis?—A Pilot Study

**DOI:** 10.3390/jcm13113150

**Published:** 2024-05-28

**Authors:** Theresa Reischer, Catherine Sklenar, Alexandra Perricos-Hess, Heinrich Husslein, Lorenz Kuessel, René Wenzl, Christine Bekos

**Affiliations:** Department of Obstetrics and Gynecology, Medical University of Vienna, 1090 Vienna, Austria; theresa.reischer@meduniwien.ac.at (T.R.); catherine.sklenar@outlook.com (C.S.); alexandra.perricos@meduniwien.ac.at (A.P.-H.); heinrich.husslein@meduniwien.ac.at (H.H.); christine.bekos@meduniwien.ac.at (C.B.)

**Keywords:** fatigue, endometriosis, laparoscopy, surgery, quality of life

## Abstract

**Background:** Fatigue is mentioned as one of the most significant symptoms of endometriosis. The impact of laparoscopic endometriosis surgeries on fatigue remains unknown. The aim of this study was to investigate, for the first time, the effect of laparoscopic surgery in endometriosis patients, with the complete removal of endometriotic lesions, on the severity of fatigue. **Methods:** This is a single-center prospective pilot study including 58 participants. Participants were recruited at the Tertiary Endometriosis Referral Center of the Medical University of Vienna between February 2020 and November 2021. Thirty patients with histologically proven endometriosis were compared to a control group of 28 patients who underwent a laparoscopy for benign gynecologic conditions other than endometriosis. All participants were interviewed using the Fatigue Severity Scale before their surgery and 6 months afterward. Relationships between variables were established using regression analysis and associations were quantified as odds ratios. **Results:** Fatigue was significantly more severe preoperatively in patients with endometriosis when compared to controls ((odds ratio (OR): 1.82; 95% confidence interval (CI): 1.24–2.67, *p* < 0.01). Six months postoperatively, the fatigue severity score of endometriosis patients decreased significantly (*p* < 0.01). In multivariate analysis, fatigue was significantly associated with endometriosis (OR: 4.50, CI: 1.14–17.8, *p* < 0.05), when adjusted for abdominal pain and menstrual bleeding. Fatigue in patients with endometriosis was not associated with disease stage or the presence of deep endometriosis. **Conclusions:** Fatigue is a frequent and bothersome symptom in patients with endometriosis. Within our study, we demonstrated for the first time that fatigue responds to surgical treatment. The management of fatigue is crucial to improving patients’ quality of life.

## 1. Introduction

Endometriosis is a common chronic disease affecting ten percent of women in their reproductive years, which can lead to infertility, adnexal masses, chronic pelvic pain, and, in rare cases, the obstruction of the bowel or urinary tract [1]. Patients with endometriosis may initially present with symptoms including dysmenorrhea, dyspareunia, chronic pelvic pain, and infertility [2]. In addition, this disease can have a significant social and psychological impact on the lives of affected women, including negative effects on quality of life, intimate relationships, planning for and having children, education, work, and emotional well-being [3]. Fatigue is another bothersome and common symptom that has been reported in several endometriosis-related studies [4,5,6].

Fatigue is defined as a subjective, persistent, individual feeling of exhaustion or tiredness, not improving with rest [7], and is associated with chronic illnesses such as rheumatoid arthritis, malignant diseases, and multiple sclerosis [8,9,10]. The prevalence of fatigue is significantly higher in patients with endometriosis, compared to the healthy female population [4]. Although chronic fatigue is mentioned as one of the most significant symptoms of endometriosis, it is still a widely neglected topic [11]. Even large studies addressing endometriosis-related symptoms miss systematically evaluating fatigue [12,13,14]. The impact of fatigue is enormous, as women’s educational performance, as well as their ability to play sports and take part in social activities, are affected [15]. 

Few studies including large populations of more than 1000 study participants reference fatigue as a symptom of endometriosis [4,5,6]. A multi-center matched case–control study, including 560 patients with endometriosis and 560 controls, demonstrated a frequency of fatigue of 50.7% of patients with endometriosis, compared to 22.4% of healthy women [16]. Fatigue was associated with endometriosis, pain, insomnia, depression, and occupational stress. In a more recent study evaluating 280 patients with a clinical diagnosis of endometriosis, 85.3% of the patients had moderate to severe fatigue [17]. The association between endometriosis and fatigue might be explained by the excessive immune response to continuous, silent inflammation determined by the presence of the disease [18]. Furthermore, chronic pain is known to worsen inflammation and subsequently result in even more fatigue [19]. Studies have also suggested that endometriotic lesions create a specific immune microenvironment resembling a tumor-like inflammatory profile [20]. Given the association between inflammatory cytokines and fatigue in cancer patients [21], it is plausible to consider that elevated cytokine levels observed in endometriosis could contribute to the development of fatigue symptoms in affected women.

Next to symptomatic treatment options, surgery remains the gold standard for women with infertility or pain resistant to medical therapy [22]. Further indications for surgical treatment are the need to exclude a malignancy of an adnexal mass, or obstruction of the bowel or urinary tract. Postoperative benefits including improved fertility and reduced pain have been shown in several studies [23,24]. A recent study evaluating patients with stage four endometriosis demonstrated a significant decrease in depression complaints and a significant increase in sleep quality six months after the operation [25].

However, it remains unclear whether laparoscopic endometriosis operations may improve fatigue symptoms in the same way as pain can be reduced by surgery. We hypothesized that, by removing endometriotic lesions and therefore altering the immune microenvironment, this could also reduce not only pain, but also fatigue. The aim of this study was to evaluate, for the first time, the effect of laparoscopic operations in endometriosis patients by using the Fatigue Severity Scale six months postoperatively. Secondary, we aimed to compare the severity of fatigue in patients with endometriosis to controls. 

## 2. Materials and Methods

### 2.1. Study Population and Study Design

The present study was conducted as a prospective pilot study at the Tertiary Endometriosis Referral Center of the Medical University of Vienna between February 2020 and November 2021. The severity of fatigue was evaluated before and six months after laparoscopic surgery in patients affected by endometriosis. Furthermore, we compared the severity of fatigue in endometriosis patients to a cohort of control patients without endometriosis.

Overall, 60 premenopausal women, 18–50 years of age, with a minimal Visual Analog Fatigue Scale (VAFS) score of 3 were included. All patients who underwent operative laparoscopy due to suspected endometriosis, infertility, chronic pelvic pain, benign adnexal masses, or uterine leiomyoma were included. Patients with suspected adenomyosis assessed by ultrasound based on the Morphological Uterus Sonographic Assessment (MUSA) criteria [26] were not included. Women with pronounced deep infiltrating endometriosis with the need for bowel resection were not included. Patients with suspected deep infiltrating endometriosis following a sonography received an additional Magnetic resonance imaging (MRI) for further confirmation and to help plan the surgery accordingly. All patients were operated on by one of the three participating surgeons. All of them are members of the certified endometriosis center and are high-volume endometriosis surgeons. 

A minimum VAFS score of 3 was chosen for the control group, to allow for a comparison of the change in fatigue severity after the intervention. The VAFS was developed based on the VAS often used to measure pain. It consists of a ten centimeter horizontal line, starting with 0, indicating “no fatigue”, and ending at 10, representing “very severe fatigue” [27]. 

While all patients with endometriosis had a Visual Analog Scale score of 3 or more, 44 patients had to be screened to include 30 patients for the control group. Two patients had to be excluded due to loss of follow-up. The case group included 30 women with diagnosed endometriosis. The control group consisted of 28 women who underwent a laparoscopic intervention due to benign gynecologic conditions (respectively, 8 myomectomies, 16 cystectomies, and 4 adhesiolysis) without anemia. No macroscopic or histological evidence of endometriosis at the time of the laparoscopy was found in the control group. None of the patients, neither from the endometriosis group nor from the control group, was taking hormonal treatment preoperatively. 

Endometriosis was diagnosed by visual inspection during surgery and was confirmed histologically. In all of the patients included in the study, all visible endometriotic lesions were removed during fertility-sparing surgery. Lesions were generally removed by excision, using either a monopolar scissor or bipolar energy device; only very small superficial lesions were ablated. If excision was used, careful dissection of the healthy surrounding tissue was performed, to ensure complete excision of the affected tissue. Endometriosis was classified intraoperatively, in accordance with the revised American Fertility Society Score (rAFS) as I (minimal), II (mild), III (moderate), or IV (severe), and reported in the operation notes and the discharge letter. In addition, completeness of the surgery removing endometriosis was reported in the operation notes (R0).

All patients had an internal medicine check-up previous to in-patient admission, including blood tests. Blood tests included full blood count, basic metabolic parameters including electrolytes, liver and kidney function test, thyroid function test, and a C-reactive protein test, as well as a virology screen for HIV, hepatitis B, and hepatitis C. Furthermore, they all had to fill in a pre-anesthesia questionnaire, which included chronic conditions and current medication. This questionnaire was discussed with an anesthesiologist at the pre-anesthesia clinic, when the consent form was signed. These reports were used to check for adherence with the inclusion and exclusion criteria. Conditions known to cause fatigue, such as known infectious diseases including SARS-CoV2-infection, chronic autoimmune disease, anemia, fibromyalgia, or malignancy, were excluded.

All participants were asked to complete two detailed questionnaires the day before surgery and at their six months postoperative follow-up visit. The period of six months was chosen to ensure recovery after surgery and have representable results on fatigue. Endometriosis patients were asked to complete an additional third questionnaire, the Endometriosis Health Profile 30 (EHP30), to assess the quality of life of these patients.

The study was planned and started before the COVID-19 pandemic. The inclusion period had to be extended due to the pandemic; otherwise, there were no major limitations due to the pandemic, but the postoperative interviews were mainly (93.1%; 54 of 58) conducted by telephone to reduce patient contact. All patients were tested for SARS-CoV-2 before inpatient admission and every 48 h during their hospital stay. There was no confirmed SARS-CoV-2 infection in any of the patients during the hospital stay. During the follow-up period, all patients were asked whether they had experienced COVID-19-related symptoms or had a confirmed SARS-CoV-2 infection, and all patients answered in the negative. 

### 2.2. Questionnaires

The pre- and postoperative interviews included three interviewer-administered questionnaires. The questionnaire regarding medical history and symptoms included demographic details, family history, and details associated with endometriosis. Furthermore, this questionnaire addressed questions regarding gynecological history, pre-existing health conditions, medication use, partnership, and fertility. The questionnaire was developed by endometriosis specialists from the Medical University of Vienna [28]. For statistical analysis, we calculated an abdominal pain score by multiplying the average Visual Analogue Scale (VAS) score of pain by days of pain. The intensity of menstrual blood loss was assessed as low (<3 pads/tampons used per day, in the first days of menstruation), normal and strong (>one pad/tampon every 2–3 h, use of several pads during the night). 

Fatigue severity was assessed by the Fatigue Severity Scale (FSS). The FSS, first described by Krupp et al., is one of the most frequently used tools to assess fatigue in patients with chronic illness [29,30]. It was initially validated for patients affected by multiple sclerosis and systemic lupus erythematosus [31]. This questionnaire consists of 9 items (questions) investigating the severity of fatigue in different situations during the last 3 months. To objectivize the severity of fatigue, patients could choose on a scale from 1 to 7, where 1 indicates strong disagreement and 7 indicates strong agreement. We used the German version translated by Valko et al. [32].

In order to be able to compare different values between the groups, the mean score on the 9 items was used as the Fatigue Severity Scale score (FSS). Previous publications suggested a categorization into non-fatigue (FSS ≤ 4.0), borderline fatigue (4.0 < FSS < 5.0), and fatigue (FSS ≥ 5.0). A fatigue score of ≥5 was considered as relevant fatigue and used as a binary parameter for further calculations [33,34,35].

In addition, to evaluate the health-related quality of life of women with endometriosis, the EHP30 was used. The EHP30 is a widely known instrument, including 30 items and five scales: pain, control and powerlessness, emotional well-being, social support, and self-image [36]. Each scale is standardized on a scale of 0–100, where 0 indicates the best health status, whereas 100 indicates the worst health status. The overall EHP score is calculated according to the recommendation of the University of Oxford; the total of the raw scores of each item is divided by the maximum possible raw score of all the items in the scale (120) and multiplied by 100 [37].

### 2.3. Ethical Approval

Informed consent was obtained from each participant prior to inclusion in the study. Ethics approval was provided by the institutional ethics committee of the Medical University of Vienna (EK 2253/2019, 14 January 2020).

### 2.4. Statistical Analysis

All statistical tests were performed using SPSS version 23.0. Data were presented either as mean ± standard deviation (SD), in the case of standard distribution, or median and interquartile range (IQR). The Shapiro–Wilk Test was used to check for normal distribution. Characteristics between the endometriosis and control groups were analyzed using Welch’s *t*-test or Fisher’s exact test; the non-parametric Mann–Whitney *U*-test and Kruskal–Wallis test were used for further comparison between groups. For paired statistics pre- and postoperatively, the Wilcoxon signed ranks test was used. Univariate logistic regression was used to assess the association between fatigue (≥5) and possible predictors (endometriosis, abdominal pain, intensity of menstrual blood loss, high body mass index (BMI), age, parity, and length of menstruation). Subsequently, a multivariate logistic regression model was stepwise fitted to test variables associated with fatigue that showed a trend with a *p*-value of <0.1 after univariate analysis. Associations were quantified as odds ratios with a 95% CI. A *p*-value of <0.05 was considered statistically significant. 

## 3. Results

In total, 30 patients with endometriosis and 28 controls without endometriosis were evaluated before and six months after surgery. The mean age of patients with endometriosis was 29.6 years, significantly lower than that of 34.3 years in the control patients (*p* < 0.01). All other characteristics did not differ significantly between the groups. Demographic data are shown in Table 1. 

The endometriosis group included 16 (53.3%) women with mild stages (rAFS Stage I or II) and 14 (46.7%) women with advanced stages (rAFS Stage III or IV). In 18 (60%) patients, peritoneal endometriosis was observed, in 13 (43%) ovarian endometriosis was observed, and in 14 (37%) deep endometriosis was observed. In four (13%) patients, we observed a combination of two lesion types, and in seven (23%), a combination of all three lesions types was observed. More details regarding disease stage and location/type of ectopic lesions are shown in the Supplementary Materials (Table S1).

### 3.1. Fatigue

The Fatigue Severity Scale score (FSS) was significantly higher in patients with endometriosis compared to controls, preoperatively (OR 1.82; 95% CI: 1.24–2.67, *p* < 0.01). Six months after surgical intervention, the FSS decreased significantly in patients affected by endometriosis, whereas there was no significant change noted in the control group. Details are shown in Table 2. 

**Figure 1:**
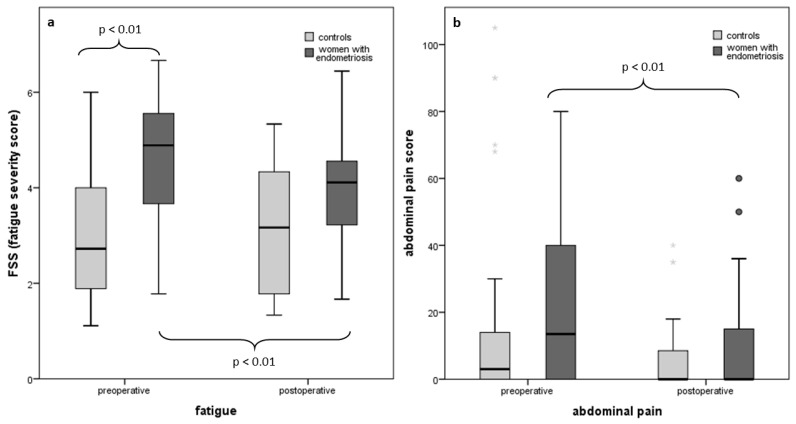
Fatigue severity score (**a**) and abdominal pain (intensity × days; (**b**)) preoperatively and six months postoperatively in patients with endometriosis and control patients without endometriosis.

Postoperatively, there was no significant difference in fatigue severity between the two groups. Seven patients in total, five of the endometriosis group and two of the control group, reported being pregnant six months postoperatively, and therefore were excluded from all analyses measuring fatigue (Figure 1). 

A multivariate logistic regression model was used to evaluate variables associated with fatigue (FSS ≥ 5). Variables with a *p*-value *p* < 0.1 after univariate analysis (endometriosis, abdominal pain, and menstrual bleeding (intensity × days)) were selected for multivariate analysis. Solely the diagnosis of endometriosis was identified as an independent risk factor for fatigue (FSS ≥ 5) preoperatively, when adjusted for abdominal pain and menstrual bleeding. Neither abdominal pain nor menstrual bleeding reached statistical significance in the multivariate logistic regression model. 

Fatigue in patients with endometriosis was not associated with disease stage or the presence of deep infiltrating endometriosis (Table 3). 

### 3.2. Symptoms 

Abdominal pain scores (intensity × days) in endometriosis patients decreased significantly postoperatively (median: 13.5 (IQR: 40) vs. 0 (IQR: 15), *p* < 0.001)). In contrast, pre- and postoperative abdominal pain scores did not improve significantly in the control group. 

Abdominal pain scores of endometriosis patients were non-significantly higher in endometriosis patients preoperatively, compared to controls (median: 13.5 (IQR: 40) vs. 3 (IQR: 14), *p* > 0.05; Figure 1). While dysmenorrhea with a VAS score of three or more did not differ significantly between endometriosis patients and control (77% vs. 57%), other characteristic symptoms of endometriosis were significantly more often reported in women with endometriosis compared to controls, preoperatively. Dyspareunia with a VAS score of three or more was reported in 60% of endometriosis patients, compared to 29% of controls (*p* < 0.05). Similarly, dyschezia was significantly more frequent in patients affected by endometriosis (40% vs. 7%). After intervention, the frequencies of these symptoms decreased in endometriosis patients, and no significant differences could be observed between the groups. An overview of symptoms and conditions experienced by women diagnosed with endometriosis and controls is summarized in the Supplementary Materials, Table S2. 

## 4. Discussion

This is the first study demonstrating a reduction in fatigue severity after surgical interventions in endometriosis patients. In addition, the significant difference in fatigue severity found in patients with endometriosis compared to the control group preoperatively improved to a similar level postoperatively compared to our control group. 

While the current treatment and management of the disease focuses more on classic symptoms [38], it is important to also address fatigue when treating patients with endometriosis. Surgery is often the preferred treatment for patients experiencing chronic pain that does not respond to medical treatments, with mechanical blockages in the bowel or urinary system, or when there’s a need to rule out cancer in ovarian masses [22]. Additionally, our data indicate that patients with endometriosis who suffer from significant fatigue may also benefit from surgical intervention.

Except for one recent study, the remaining investigations evaluating the effects of surgical interventions focused on pain, fertility, and recurrence rates. One prospective cohort study evaluating patients with stage IV endometriosis demonstrated a significant decrease in depression complaints and a significant increase in sleep quality six months after the operation [25]. However, none of these studies investigated the effects of surgery on fatigue. 

The association between endometriosis and fatigue (FSS ≥ 5) remained significant after controlling for confounding factors (abdominal pain and menstrual bleeding). These data are in line with a previous publication [16] and further support an independent effect of endometriosis which cannot be attributed to disease symptoms. Therefore, endometriosis-related fatigue should be addressed in patient care. 

Fatigue in patients with endometriosis was not associated with the disease stage or the presence of deep infiltrating endometriosis. This is in line with previous data comparing patients with endometriosis and controls [16], showing that fatigue is independent of endometriosis stages. In addition, in a multivariate analysis, only a minimal association was observed between the endometriosis stage and the severity of pelvic symptoms [39]. This leads to the assumption that complex mechanisms are involved in the development of fatigue in endometriosis patients. However, previous publications also investigated a contrary association: a retrospective study, evaluating 341 infertile women with endometriosis and 332 infertile women with a normal pelvis, showed that fatigue was more common among late-stage endometriosis patients than in those with early-stage endometriosis [40]. Given that this investigation had a retrospective design and fatigue was evaluated using yes/no answers instead of validated questionnaires, these results seem to be less reliable. 

Another result underlining the benefits of surgery in this patient cohort is that quality of life, measured using the EHP-30, improved six months postoperatively in endometriosis patients. This is in line with previous data from a cohort study, including 62 women who had undergone laparoscopic excision of endometriosis for pelvic pain, which showed that all five scales of the EHP-30 improved at four weeks postoperatively and that this effect persisted for up to 6.8 years in follow-ups [41]. 

While the current treatment and management of the disease focus more on classic symptoms [38], it is important to also address fatigue when treating patients with endometriosis. Surgery is preferred in patients who suffer from chronic pain resistant to medical treatment, with mechanical obstructions of the bowel or the urinary system, or to exclude malignancy in ovarian masses [22]. Our data suggest that endometriosis patients suffering from significant fatigue might also benefit from surgery. Next to surgery, physical but also psychological interventions were shown to be effective in treating fatigue. Light- and moderate-intensity physical activities appear to achieve the greatest reductions in cancer-related fatigue, whereas vigorous activity may exacerbate the condition [42]. In addition, physical activity is known to reduce pain [43], but also to improve the quality of life and to alleviate the negative effects that patients with breast cancer suffer as a result of the treatments received [44]. Although, to date, no evidence exists for these interventions in endometriosis patients, they might be beneficial for endometriosis patients suffering from fatigue. In addition, small-group psychoeducational sessions have been demonstrated to be effective in reducing fatigue [45]. 

The applications of this study may be limited by the design of the study, which does not allow us to evaluate causal effects. In addition, we were not able to evaluate additional confounding factors including insomnia and sleep disturbance. Another weakness of our study is that patients were evaluated six months postoperatively, and, therefore, long-term follow-up data are not available. Furthermore, few patients were taking hormonal treatment postoperatively, which could alter the outcomes including menstrual bleeding, pain, and fatigue. Due to the small number of patients, subgroup analysis for these cases was not feasible. 

The strengths of our study include that the operations were performed by the same team with the same treatment methods in the same group of patients. A comparison was made in the preoperative and postoperative periods and a control group was included. In addition, our study had a prospective design. Another strength is that none of our patients suffered from confirmed SARS-CoV-2 infections or reported symptoms suspicious for COVID-19 during the observation period, which excludes another possible confounding factor. A thorough patient history with comorbidities (including depression, fibromyalgia, or irritable bowel disease) and medication that might have an impact on fatigue was performed, and affected patients were excluded. Our drop-out rate of only two patients was very low, strengthening the results provided. 

## 5. Conclusions

This prospective pilot study demonstrates, for the first time, that surgery not only seems to improve pain but also the quality of life and reduces fatigue in women with endometriosis. Further studies are needed to evaluate the effects of surgery on fatigue in such patients. These results are the basis for planning of future large-scale studies to validate these findings. In future, treatment decisions aimed at improving quality of life should emphasize surgery for effectively reducing fatigue, alongside managing chronic pain and infertility.

## Figures and Tables

**Table 1:** Characteristics of patients with endometriosis and controls; data are either demonstrated in absolute values and percentages or mean and standard deviation.

Characteristics	Endometriosis (N = 30)	Controls (N = 28)	*p*-Value
**Age**	29.6 (±4.4)	34.3 (±6.8)	*p* < 0.01
**BMI**	24.6 (±5.5)	23.3 (±6.5)	n.s.
**Ethnicity**			
Caucasian	26 (87%)	28(100%)	n.s.
Non-Caucasian	4 (13%)	0 (0%)	
**Patients with a child/children**			
Yes	8 (27%)	9 (32%)	n.s.
no	22(73%)	19 (68%)	
**Wish for child**			
Yes	28 (93%)	20 (71%)	n.s.
No	2 (7%)	8 (29%)	
**Postoperative pregnancy**			
Yes	5 (17%)	2 (7%)	n.s.
No	25 (83%)	26 (93%)	

**Table 2:** Fatigue severity score (FSS) in women with endometriosis and controls.

	Endometriosis	Controls	*p*-Value
**FSS Median (IQR)**			
preoperative	4.8 (IQR 2.6)	2.7 (IQR 2.2)	*p* < 0.01
postoperative	4.1 (IQR 2.3)	3.2 (IQR 2.8)	n.s.
**FSS ≥ 5; N (%)**			
preoperative	12/30 (40%)	4/28 (14.3%)	*p* < 0.05
postoperative	4/25 (16%)	3/26 (11.5%)	n.s.

**Table 3:** Univariate and multivariate statistics for factors associated with fatigue (FSS ≥ 5) prior to operation.

Predictor	OR (95% CI)	*p*-Value
**Univariate Analysis**		
**Endometriosis**	5.08 (1.35–19.06)	*p* < 0.01
**Abdominal Pain**	1.01 (1.00–1.02)	*p* < 0.1
**Menstrual Bleeding (Intensity × Days)**	1.03 (0.96–1.10)	*p* < 0.1
**High BMI (** **≥** **25)**	2.25 (0.65–7.80)	n.s.
**Age**	0.94 (0.85–1.03)	n.s.
**Parity**	0.46 (0.12–1.74)	n.s.
**Multivariate Analysis**		
**Endometriosis**	4.50 (1.14–17.8)	*p* < 0.05

## Data Availability

The dataset is available on request from the authors.

## Supplementary Materials

The following supporting information can be downloaded at: https://www.mdpi.com/article/10.3390/jcm13113150/s1. Table S1. Endometriosis patient characteristics for disease stage and ectopic lesions; Table S2. Pre- and postoperative symptoms in endometriosis patients and controls.
