# Supplementary material for: Can Laparoscopic Surgery Reduce Fatigue in Women with Endometriosis?—A Pilot Study
Source: J Clin Med. 2024 May 28;13(11):3150. doi: 10.3390/jcm13113150 (PMC11172980; doi:10.3390/jcm13113150)
Supplement: Supplementary file 1 [file jcm-13-03150-s001.zip › jcm-3001460-supplementary.pdf]

Journal of
Clinical Medicine

MDPI

Supplementary Materials

Table S1. Endometriosis patient characteristics for disease stage and ectopic lesions;

rAFS stage of endometriosis, N (%)	
I, minimal	12 (40%)
II, mild	4 (13.3%)
III, moderate	2 (6.7%)
IV, severe	12 (40%)
lesion type, N (%)	
peritoneal	18 (60%)
ovarian	13 (43%)
deep endometriosis	14 (37%)
combination of two lesion types	4 (13%)
combination of three lesion types	7 (23%)

Table S2. Pre and postoperative symptoms in endometriosis patients and controls;

	Endometriosis N (%)	*Controls N (%)*	*p-value*
Abdominal pain ≥ 3			
preoperative	15 (50%)	13 (46%)	n.s.
postoperative	11 (37%)	8 (29%)	n.s.
Dysmenorrhea ≥ 3			
preoperative	23 (77%)	16 (57%)	n.s.
postoperative	17 (57%)	16 (57%)	n.s.
Dyspareunia ≥ 3			
preoperative	18 (60%)	8 (29%)	p<0.05
postoperative	7 (23%)	5 (18%)	n.s.
Dysuria ≥ 3			
preoperative	6 (20%)	1 (4%)	p<0.05
postoperative	3 (10%)	1 (4%)	n.s.
Dyschezia ≥ 3			
preoperative	12 (40%)	2 (7%)	p<0.01
postoperative	4 (13%)	0 (0%)	n.s.
